# Supplementary figures and images for: Does cardiorespiratory fitness mediate or moderate the association between mid-life physical activity frequency and cognitive function? findings from the 1958 British birth cohort study
Source: PLoS One. 2024 Jun 7;19(6):e0295092. doi: 10.1371/journal.pone.0295092 (PMC11161044; doi:10.1371/journal.pone.0295092)

# **Supplementary figure 1. Sample flow diagram**


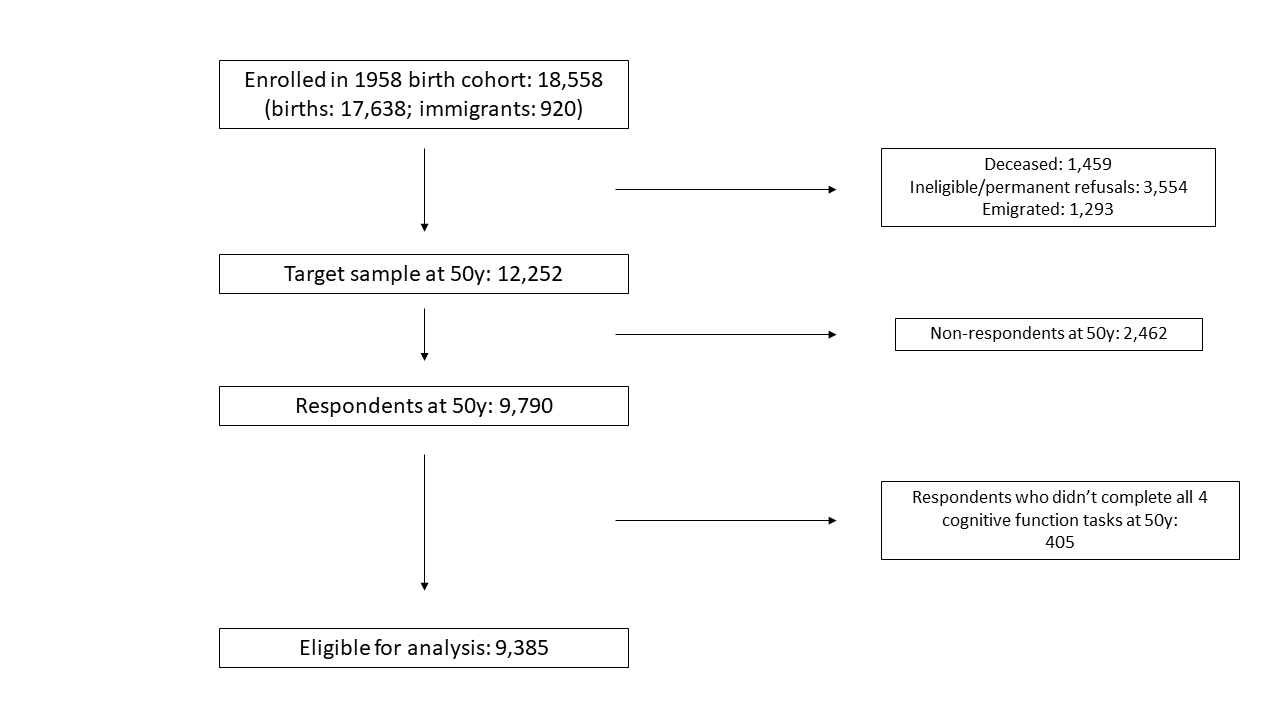

Supplement: S1 Fig — (DOCX) [file pone.0295092.s001.docx]
