# Supplementary material for: Does cardiorespiratory fitness mediate or moderate the association between mid-life physical activity frequency and cognitive function? findings from the 1958 British birth cohort study
Source: PLoS One. 2024 Jun 7;19(6):e0295092. doi: 10.1371/journal.pone.0295092 (PMC11161044; doi:10.1371/journal.pone.0295092)
Supplement: S2 Fig — (DOCX) [file pone.0295092.s002.docx]

# **Supplementary figure 2. Directed acyclic graph (DAG)***


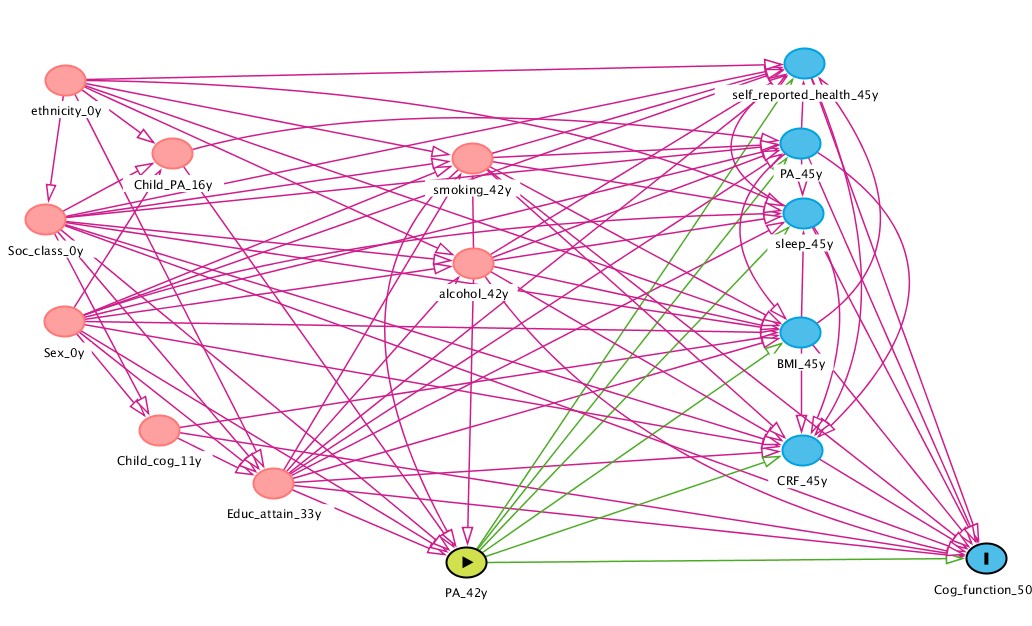


*Based on the above DAG, the minimum adjustment set for the direct effect of physical activity (PA_42y) on cognitive function (Cog_function_50) included: social class at birth (S*oc_class_0y*), childhood cognitive function (11y) (C*hild_cog_11y*), sports participation (16y) (Child_PA_16y), educational attainment (33y) (*Educ_attain_33y*), smoking status (42y) (s*moking_42y*), alcohol consumption (42y) (*alcohol_42y*), BMI (45y) (*BMI_45y*), physical activity level (45y) (*PA_45y*), Sleep (45y) (*sleep_45y*) and self-rated health in previous 12 months (45y) (*self_rated_health_45y*). Note that this DAG illustrates 45y BMI and 45y PA (amongst others) as intermediate confounders of the 42y PA ꟷ 50y cognitive function association; this *causal* diagram, which aims to illustrate the underlying causal network underpinning the relationship between 42y PA and 50y cognitive function, is distinct from the *prediction* equation for NETCRF described in the methods.
