# Supplementary material for: Does cardiorespiratory fitness mediate or moderate the association between mid-life physical activity frequency and cognitive function? findings from the 1958 British birth cohort study
Source: PLoS One. 2024 Jun 7;19(6):e0295092. doi: 10.1371/journal.pone.0295092 (PMC11161044; doi:10.1371/journal.pone.0295092)
Supplement: S1 Table — (DOCX) [file pone.0295092.s003.docx]

# **Supplementary Table 1. Definitions of causal estimators for a continuous outcome (overall cognition z-score) with a binary exposure (*PA at 42y*) and continuous mediator (*NETCRF at 45y*)**

| **Estimated causal effect** | **Formula** | **Interpretation** |
| --- | --- | --- |
| _e_OE | E(Ya,G_a\|v_ \| v) - E(Ya*,G_a*\|v_ \| v) | The difference in cognition between being physical activity≥1/week and <1/week, with NETCRF taking a random value from the distribution when physical activity≥1/week and physical activity<1/week respectively. Pathways involving and not involving NETCRF are represented. |
| _e_CDE | E(Ya,m* \| v) – E(Ya*,m* \|v) | The direct effect comparing physical activity≥1/week versus <1/week when NETCRF in both cases is set to the mean value taken when physical activity is <1/week (conditional on the covariates). This is the effect due to pathways that do not involve NETCRF (i.e., no mediation or interaction by NETCRF). |
| _e_rPNIE | E(Ya*, G_a\|v_ \| v) – E(Ya*, G_a*\|v_ \|v) | The difference in cognition when activity<1/week but NETCRF takes a random value from the distribution when physical activity≥1/week vs physical activity<1/week (conditional on covariates). This is the effect only due to mediation and does not involve interaction. |
| _e_rINTREF | [E(Ya,G_a*\|v_ \| v) – E(Ya*,G_a_*_\|v_ \| v)] – [E(Ya,m* \| v) – E(Ya*,m* \|v)] | The effect of PA on cognition due to the interaction between PA and NETCRF that operates when NETCRF is set to its mean value when physical activity is <1/week (i.e., *the effect of PA on cognition due solely to the interaction between PA and NETCRF)*. |
| _e_rINTMED | [E(Ya,G_a\|v_ \| v) – E(Ya,G_a*\|v_ \| v)] – [E(Ya*,G_a\|v_ \| v) - E(Ya*,G_a*\|v_ \| v)] | The effect of PA on cognition due to the interaction between PA and NETCRF and the fact that PA is causing high NETCRF *(i.e., the effect due to both mediation and interaction).* |

_e_OE, overall effect; _e_CDE, controlled direct effect; _e_rPNIE, randomized analogue of pure natural indirect effect; _e_rINTREF, randomized analogue of reference interaction; _e_rINTMED, randomized analogue of mediated interaction; Y= cognition; m* is the value at which NETCRF is controlled at (i.e., the mean value taken when physical activity is <1/week). G_a|v_= a random draw from the distribution of NETCRF amongst those with PA at level *a* conditional on covariates v; a and a∗ are physical active ≥1/week and <1/week respectively.

# 
