# Supplementary material for: Does cardiorespiratory fitness mediate or moderate the association between mid-life physical activity frequency and cognitive function? findings from the 1958 British birth cohort study
Source: PLoS One. 2024 Jun 7;19(6):e0295092. doi: 10.1371/journal.pone.0295092 (PMC11161044; doi:10.1371/journal.pone.0295092)
Supplement: S2 Table — (DOCX) [file pone.0295092.s004.docx]

# **Supplementary Table 2. Scores on individual cognitive function tasks at 50y**

| Cognitive function task* | Males | Females |
| --- | --- | --- |
| Immediate verbal memory (Number of words correctly recalled) | 6.4 (1.5) | 6.7 (1.5) |
| Verbal fluency (Number of animals mentioned) | 22.3 (6.4) | 22.3 (6.2) |
| Visual processing speed (Total number of letters scanned) | 313 (270, 375) | 326 (284, 402) |
| Delayed verbal memory (Number of words correctly recalled after delay) | 5.2 (1.8) | 5.6 (1.8) |

*Summarised as mean(SD) or median(25^th^, 75^th^ centile)
