# Supplementary material for: Does cardiorespiratory fitness mediate or moderate the association between mid-life physical activity frequency and cognitive function? findings from the 1958 British birth cohort study
Source: PLoS One. 2024 Jun 7;19(6):e0295092. doi: 10.1371/journal.pone.0295092 (PMC11161044; doi:10.1371/journal.pone.0295092)
Supplement: S3 Table — (DOCX) [file pone.0295092.s005.docx]

# **Supplementary Table 3. Sample characteristics of original 1958 cohort* vs analytical sample (n=9,385), at birth and in early life**

|  | N(%) / Mean(SD) | | | |
| --- | --- | --- | --- | --- |
| Variable (reporting age (y)) | Males | | Females | |
|  | Original 1958 cohort | Analytical sample | Original 1958 cohort | Analytical sample |
| Social class (birth)^Ϯ^ |  |  |  |  |
| *Professional/managerial* | 1,585 (17.3) | 783 (20.1) | 1,474 (17.2) | 774 (19.2) |
| *Skilled non-manual* | 869 (9.5) | 393 (10.1) | 796 (9.3) | 396 (9.8) |
| *Skilled manual* | 4,507 (49.1) | 1,836 (47.2) | 4,179 (48.7) | 1,942 (48.1) |
| *Partly skilled/Unskilled/Other* | 2,223 (24.2) | 879 (22.6) | 2,140 (24.9) | 925 (22.9) |
| Childhood cognition score (11y) | 16.4 (8.1) | 17.5 (7.8) | 16.2 (7.6) | 17.2 (7.3) |
| Sports participation (16y) |  |  |  |  |
| *No chance* | 113 (1.9) | 54 (1.6) | 260 (4.6) | 159 (4.5) |
| *Hardly ever* | 716 (12.2) | 413 (12.3) | 1,980 (36.1) | 1,203 (34.3) |
| *Sometimes* | 1,906 (32.5) | 1,075 (32.1) | 2,116 (38.6) | 1,372 (39.1) |
| *Often* | 3,139 (53.4) | 1,808 (54.0) | 1,129 (20.6) | 1. (22.0) |

^*^Maximal sample at: birth= 17,773; 11y= 14,131; 16y= 11,359; ^†^Recorded at birth or at 7y if missing at birth;
