# Supplementary material for: Does cardiorespiratory fitness mediate or moderate the association between mid-life physical activity frequency and cognitive function? findings from the 1958 British birth cohort study
Source: PLoS One. 2024 Jun 7;19(6):e0295092. doi: 10.1371/journal.pone.0295092 (PMC11161044; doi:10.1371/journal.pone.0295092)
Supplement: S4 Table — (DOCX) [file pone.0295092.s006.docx]

# **Supplementary Table 4. E-values for estimated overall and controlled direct effects (expressed on risk ratio scale)**

|  | Males | | Females | |
| --- | --- | --- | --- | --- |
| Causal estimate | RR* (95% CI) | E-value of RR (E-value for limit of CI closest to null) | RR* (95% CI) | E-value of RR (E-value for limit of CI closest to null) |
| _e_OE | 1.11 (1.05, 1.18) | 1.47 (1.29) | 1.05 (0.99, 1.11) | 1.27 (1) |
| _e_CDE | 1.11 (1.05, 1.18) | 1.46 (1.27) | 1.03 (0.97, 1.10) | 1.21 (1) |

_e_OE, estimated overall effect; _e_CDE, estimated controlled direct effect; *Transformed into risk ratios using the transformation described by Vanderweele et al. (2017).
