# Supplementary material for: Does cardiorespiratory fitness mediate or moderate the association between mid-life physical activity frequency and cognitive function? findings from the 1958 British birth cohort study
Source: PLoS One. 2024 Jun 7;19(6):e0295092. doi: 10.1371/journal.pone.0295092 (PMC11161044; doi:10.1371/journal.pone.0295092)
Supplement: S7 Table — (DOCX) [file pone.0295092.s009.docx]

# **Supplementary Table 7. Estimated overall, controlled direct and randomised analogues of the pure natural indirect, mediated and interaction effects of physical activity frequency at 42y on visual processing speed at 50y (mediated/moderated by NETCRF at 45y)**

|  | Difference in mean visual processing speed z-score (95% CI) | |
| --- | --- | --- |
|  | Physical activity (42y; *ref: <once/week*) | |
|  | Males (n=4,614) | Females (n=4,771) |
| _e_Overall effect (OE) | 0.06 (-0.02, 0.11) | 0.07 (-0.01, 0.14) |
| _e_Controlled Direct Effect (CDE) | 0.06 (-0.02, 0.13) | 0.08 (-0.01, 0.16) |
| _e_Randomized analogue of Pure Natural Indirect Effect (rPNIE) | 0.00 (-0.03, 0.04) | 0.02 (-0.01, 0.06) |
| _e_Randomized analogue of Reference Interaction (rINTREF) | -0.00 (-0.00, 0.00) | -0.00 (-0.01, -0.00) |
| _e_Randomized analogue of Mediated Interaction (rINTMED) | -0.00 (-0.03, 0.01) | -0.02 (-0.04, -0.01) |
