# Supplementary material for: Does cardiorespiratory fitness mediate or moderate the association between mid-life physical activity frequency and cognitive function? findings from the 1958 British birth cohort study
Source: PLoS One. 2024 Jun 7;19(6):e0295092. doi: 10.1371/journal.pone.0295092 (PMC11161044; doi:10.1371/journal.pone.0295092)
Supplement: S1 Text — (DOCX) [file pone.0295092.s011.docx]

**Supplementary material: Does cardiorespiratory fitness mediate or moderate the association between mid-life physical activity frequency and cognitive function? Findings from the 1958 British birth cohort study**

Norris, T.^1^, Mitchell, J.J.^1^, Blodgett, J.M.^1^, Hamer, M.^1^ & Pinto Pereira, S.M.^1^

^1^Institute of Sport, Exercise and Health, Division of Surgery and Interventional Science, Faculty of Medical Sciences, UCL, London, UK

Contents

[**Supplementary text S1. Variable derivation** 2](#_Toc165543535)

[**Physical activity frequency variable (42y)** 2](#_Toc165543536)

[**Potential confounders** 2](#_Toc165543537)

[**Supplementary figure 1. Sample flow diagram** 4](#_Toc165543538)

[**Supplementary figure 2. Directed acyclic graph (DAG)*** 5](#_Toc165543539)

[**Supplementary Table 1. Definitions of causal estimators for a continuous outcome (overall cognition z-score) with a binary exposure (*PA at 42y*) and continuous mediator (*NETCRF at 45y*)** 7](#_Toc165543540)

[**Supplementary Table 2. Scores on individual cognitive function tasks at 50y** 8](#_Toc165543541)

[**Supplementary Table 3. Sample characteristics of original* vs included sample (n=9,385), at birth and in early life** 9](#_Toc165543542)

[**Supplementary Table 4. E-values for estimated overall and controlled direct effects (expressed on risk ratio scale)** 10](#_Toc165543543)

[**Supplementary Table 5. Estimated overall, controlled direct and randomised analogues of the pure natural indirect, mediated and interaction effects of physical activity frequency at 42y on immediate verbal memory at 50y (mediated/moderated by NETCRF at 45y)** 11](#_Toc165543544)

[**Supplementary Table 6. Estimated overall, controlled direct and randomised analogues of the pure natural indirect, mediated and interaction effects of physical activity frequency at 42y on verbal fluency at 50y (mediated/moderated by NETCRF at 45y)** 12](#_Toc165543545)

[**Supplementary Table 7. Estimated overall, controlled direct and randomised analogues of the pure natural indirect, mediated and interaction effects of physical activity frequency at 42y on visual processing speed at 50y (mediated/moderated by NETCRF at 45y)** 13](#_Toc165543546)

[**Supplementary Table 8. Estimated overall, controlled direct and randomised analogues of the pure natural indirect, mediated and interaction effects of physical activity frequency at 42y on delayed verbal memory at 50y (mediated/moderated by NETCRF at 45y)** 14](#_Toc165543547)

[**References** 15](#_Toc165543548)

# **Supplementary text S1. Variable derivation**

## **Physical activity frequency variable (42y)**

During face-to-face interviews at 42y, participants were shown a card with a list of physical activities on (‘competitive sport of any kind’, ‘keep fit or aerobics classes’, ‘running or jogging’, ‘swimming’, ‘cycling’, ‘walks’, ‘water sports’, ‘outdoor sports’, ‘dancing’, ‘any other sport or leisure activity which involves physical exercise’). They were then asked: “Do you regularly take part in any of the activities on this card? By regularly I mean at least once a month, for most of the year”. If participants responded ‘yes’, they were then asked: “How often do you take part in any activity of this type?”, with available responses: ‘≤2-3 x per month’; ‘1 x per week’; ‘2-3 x per week’; ‘4-7 x per week’. From these responses we derived a dichotomous variable representing whether people participated in physical activity ‘less than once per week’ (0) or ‘at least once per week’ (1).

## **Potential confounders**

Social class at birth (or at 7y if missing at birth) was defined according to the Registrar general’s classification and included the following categories: professional/managerial, skilled non-manual, skilled manual, partly skilled and unskilled/other. Cognitive function in childhood was calculated as the mean score obtained on a reading comprehension and maths test administered by schoolteachers at 11y. The reading comprehension test consisted of 35 sentences (1 mark per sentence) in which the child was required to read a sentence and choose from a selection of 5 words the most appropriate to complete the sentence. From the list, the child was required to underline the missing item which completed the sentence. The maths test consisted of 40 items (1 mark per item). The test included number skills, fractions, measures and geometry. Most questions were calculated directly, with a few involving multiple-choice answers. Further details of these assessments can be found on the CLOSER website(1). Self-reported leisure time participation in sports was measured at age 16y and categorised as: no chance, hardly ever, sometimes, often. Educational attainment was defined as the highest academic qualification obtained by age 33y. Smoking status (42y) was categorised as never, ex-smoker, current smoker. Alcohol consumption frequency (42y) was categorised into four groups: never, rarely, 2/3/4 times per month, at least twice per week. Height and weight were measured at 45y by trained nurses and BMI (kg/m^2^) calculated. Physical activity (45y) was based on adherence to contemporaneous guidelines assessed using the EPIC-PAQ and classified into three groups: inactive during leisure-time; active, but not meeting the guidelines; meeting the guidelines of at least 150 minutes per week of moderate-intensity or 75 minutes per week of vigorous-intensity PA. A sleep problems score at 45y was derived from the Clinical Interview Schedule-revised (CIS-R), with scores ranging from 0-4, with higher scores representing more problems. Self-rated health over the previous 12 months (45y) was categorised as: excellent, good, fair, poor.

# **References**

1. CLOSER. Cognitive measures in the 1958 National Child Development Study 2022 [Available from: <https://closer.ac.uk/cross-study-data-guides/cognitive-measures-guide/ncds-cognition/>.
