# Supplementary material for: Does cardiorespiratory fitness mediate or moderate the association between mid-life physical activity frequency and cognitive function? findings from the 1958 British birth cohort study
Source: PLoS One. 2024 Jun 7;19(6):e0295092. doi: 10.1371/journal.pone.0295092 (PMC11161044; doi:10.1371/journal.pone.0295092)
Supplement: Attachment — Submitted filename: v3 4WayDecompRR.docx [file pone.0295092.s012.docx]

**Reviewer #1**

Thank you for the opportunity to review this interesting, novel and potentially important study investigating the potential mediating and moderating effect of fitness in the association between physical activity and cognitive function in middle-aged adults. The link between physical activity and cognitive function is not clear-cut and studies investigating potential pathways are essential. The study includes a relatively large (n=9,385) sample and uses a novel approach to investigate mediation and/or moderation through fitness. The statistical methods used in the study are appropriate and are well explained in the text. The authors have used appropriate measures to account for missingness and potential confounding, as these are common issues in epidemiological research. In the results and the discussion, the findings are clearly presented, and potential explanations are well explored. I have only some minor comments that I hope the authors will consider in a potential revision.

*Response: Thank you for acknowledging that our study is interesting, novel and potentially important and that the statistical methods used are appropriate and well explained. Below we respond to all comments raised.*

Abstract
- Please present the proportion of women in the study. This is highly relevant as the authors present sex-specific findings.

*Response: We have edited the methods of the abstract to state: “…and overall cognitive function (outcome) at 50y were obtained from 9,385 participants (50.8% female) in the 1958 British birth cohort study.”*

Introduction
- The sentence “In the United Kingdom (UK), there are no effective treatments to reverse or delay dementia progression” makes it sound as if there are effective treatments for dementia in other countries. The lack of effective treatments for dementia is a worldwide issue, and I think this sentence understates how crucial it is to look elsewhere (modifiable risk factors). I would suggest rephrasing the sentence, either to just state that there are no effective treatments anywhere, or clarify otherwise.

*Response: This sentence now reads (page 3): “While Lecanemab has very recently been approved in the US and Japan for the treatment of Alzheimer’s disease, in the United Kingdom (UK), there remains no effective treatments to reverse or delay dementia progression.”*

- The authors state: “Additionally, CRF may also moderate the PA―cognition association, such that associations differ depending on fitness levels as higher fitness would enable participation in greater volumes/intensity of PA.” To me, the last part of the sentence sounds more like mediation (i.e. CRF -> PA -> cognitive function) rather than moderation (the association of PA with cognitive function differing by level of CRF). In my opinion, this sentence needs clarification/rephrasing.
*Response: The sentence now reads (page 3): “Thus, in addition to CRF potentially mediating the PA―cognition association, CRF may also moderate this association, with the association differing depending on fitness levels.”*

Methods
- Outcome: please include the (average) time between the initial immediate verbal memory test and the delated verbal memory test.
*Response: We have added the following sentence (page 6): “This delayed memory recall task was done approximately five minute after the initial recall task.”*

- Statistical analysis:
o Some of the variables, including important exposure variables, had a quite high missing proportion. The authors have performed multiple imputation, but it could also be informative if the authors performed complete case analyses (sensitivity) so that the reader can better understand the potential bias caused by the high missingness.

*Response: Thank you for this comment on which we have reflected at length. Having worked with this cohort extensively, we believe the data are missing at random but not completely at random (see Sterne et al for methodological details;* [*https://doi.org/10.1136/bmj.b2393*](https://doi.org/10.1136/bmj.b2393)*). Previous publications in the 1958 birth cohort support this assumption, for example, Atherton et al. showed that childhood internalizing and externalizing behaviours and cognitive ability predict missingness in follow-up (see: Journal of Epidemiology & Community Health. 2008;62(3):216-23) and see also Journal of Clinical Epidemiology, 2021; 136:44-54 [DOI:10.1016/j.jclinepi.2021.02.019]. Thus, as the reviewer rightly points out, analyses based on complete cases may be biased. For this reason, we believe that a complete case analysis would be misleading (and potentially confusing for readers). Hence, we have chosen not to show a complete case analysis in the publication.*

*Instead, we choose to focus on findings based on imputation following recommended guidelines, which we now explain in greater detail in the text (page 7): “To address missingness in data presented in Table 1, we used multiple imputation by chained equations following recommended guidelines(30). We assume the data are missing-at-random and strengthen this assumption by including in the imputation models all variables described above (i.e., 42y PA, 45y NETCRF, 50y cognitive function and potential confounders), as well as childhood internalizing and externalizing behaviours and cognitive ability which have been shown to predict missingness in follow-up(17). The number of imputations (n=30) required to achieve convergence of parameter estimates was determined as 100×fraction missing information(31).”*

*Nonetheless, we do acknowledge missingness in our dataset (see Table 1) and that it ranges from 5.5% (physical activity, smoking status and alcohol consumption at 42y, females) to 38.9% (NETCRF at 45y, females). We present below a replication of Table 2 based on a complete cases analysis. Note that minimal differences are observed in point estimates (and 95% CIs) when based on complete case or imputed data.*

**Associations between Physical activity frequency, NETCRF and overall cognitive function (complete case analysis^†^)**

|  | Mean difference in NETCRF (METS)(45y)  (95% CI) | | Mean difference in overall cognition function z-score (50y)  (95% CI) | | |
| --- | --- | --- | --- | --- | --- |
|  | Model A | Model B | Model A | Model B | Model C** |
|  | Males | | | | |
| Physical activity frequency at 42y *(ref: <once per week)* | 0.53 (0.44, 0.62) | 0.45 (0.33, 0.57) | 0.15 (0.11, 0.19) | 0.05 (-0.00, 0.10) |  |
| NETCRF at 45y (METS) |  |  | 0.04 (0.02, 0.06) | -0.00 (-0.03, 0.02) | 0.02 (-0.03, 0.07) |
|  | Females | | | | |
| Physical activity frequency at 42y *(ref: <once per week)* | 0.61 (0.51, 0.71) | 0.52 (0.40, 0.65) | 0.11 (0.07, 0.15) | 0.02 (-0.03, 0.07) |  |
| NETCRF at 45y (METS) |  |  | 0.05 (0.03, 0.07) | 0.01 (-0.01, 0.03) | 0.01 (-0.04, 0.07) |

^NETCRF: Non-exercise testing cardiorespiratory fitness; METS: metabolic equivalent values; CI: confidence interval.* Model A: unadjusted; Model B: adjusted for baseline confounders (socioeconomic position at birth, sports participation at 16y, cognitive function at 11y, academic attainment at 33y, smoking status at 42y, alcohol consumption at 42y); Model C: Model B + physical activity at 42y, BMI at 45y, physical activity at 45y, general health at 45y and sleep score at 45y; **association between PA at 42y and total cognition z-score was not adjusted for NETCRF as this would be adjusting for the potential mediator/moderator which would attenuate the overall effect of physical activity which is the estimand of interest here^

^†Sample size varies across sexes and depending on covariates included in each complete-case analysis.^

o Have the authors considered using inverse probability weighting to account for non-response and potential bias due to skewed participation (e.g. related to education)?

*Response: As explained above, we chose to impute data to retain the sample size and reduce potential bias due to non-response in the defined analytical sample. Using inverse probability weighting, in addition to imputation, would enable a re-weighting of our analytical sample back to the original cohort at birth. This is a good recommendation from the review and one that we initially considered before analysis. However, on balance, we considered our 4-way decomposition analysis to already be extremely complex; adding another layer over it (reweighting) would be both computationally challenging and intensive and unnecessarily complicated for readers. We believe the latter is true because, in mid-adulthood, participants remain broadly representative of the original study sample (see Atherton et al Journal of Epidemiology & Community Health. 2008;62(3):216-23). We also now note that, in the complete case analysis shown above, the complete case and imputed estimates differ very little, suggesting bias due to sample selection is minimal.*

*Nonetheless, we now also provide a comparison of the original and analytical samples. This is presented in (new) table S3 (shown below) and commented on in the first paragraph of the results where we now state: “Our analytical sample was broadly comparable to the 1958 cohort participating in early life, with small differences in the analytical sample being more likely to be from a higher social class, have higher cognition scores at 11y and report higher levels of PA at 16y (S3 Table).”.*

**Supplementary Table 3. Sample characteristics of original* vs included sample (n=9,385), at birth and in early life**

|  | N(%) / Mean(SD) | | | |
| --- | --- | --- | --- | --- |
| Variable (reporting age (y)) | Males | | Females | |
|  | Original sample | Included sample | Original sample | Included sample |
| Social class (birth)^Ϯ^ |  |  |  |  |
| *Professional/managerial* | 1,585 (17.3) | 783 (20.1) | 1,474 (17.2) | 774 (19.2) |
| *Skilled non-manual* | 869 (9.5) | 393 (10.1) | 796 (9.3) | 396 (9.8) |
| *Skilled manual* | 4,507 (49.1) | 1,836 (47.2) | 4,179 (48.7) | 1,942 (48.1) |
| *Partly skilled/Unskilled/Other* | 2,223 (24.2) | 879 (22.6) | 2,140 (24.9) | 925 (22.9) |
| Childhood cognition score (11y) | 16.4 (8.1) | 17.5 (7.8) | 16.2 (7.6) | 17.2 (7.3) |
| Sports participation (16y) |  |  |  |  |
| *No chance* | 113 (1.9) | 54 (1.6) | 260 (4.6) | 159 (4.5) |
| *Hardly ever* | 716 (12.2) | 413 (12.3) | 1,980 (36.1) | 1,203 (34.3) |
| *Sometimes* | 1,906 (32.5) | 1,075 (32.1) | 2,116 (38.6) | 1,372 (39.1) |
| *Often* | 3,139 (53.4) | 1,808 (54.0) | 1,129 (20.6) | 771 (22.0) |

^*^Maximal sample at: birth= 17,773; 11y= 14,131; 16y= 11,359; ^†^Recorded at birth or at 7y if missing at birth;

*Additionally, we now also acknowledge the limitations of our approach and suggest alternatives in the discussion (penultimate paragraph, page 20): “As in all longitudinal studies, loss to follow-up occurred and while participants in mid-adulthood were broadly representative of the original population, the most disadvantaged were least likely to remain[17].* While statistical approaches such as inverse probability weighting and/or the multiple imputation approach adopted in this manuscript could address potential bias due to non-response, attrition and sample selection*[64]*, efforts should also be made to re-engage all cohort members with the study*[17].* *Finally, the 1958 birth cohort is predominantly of White British ethnicity (~98% at 45y). For the above reasons, generalisability (e.g., to other ethnic groups, less fit middle-aged men and women) should be inferred with caution.”*

Results
- Please include explanations of abbreviations in the tables.
*Response: We have edited all tables to have footnotes for all abbreviations used.*

- The authors present a list of potential confounders (also illustrated in the DAG in the supplementary) in the methods section. However, several of these potential confounders are not listed in Table 2 (model B or C). For instance, BMI and sleep problems are missing. Is this a typo, or where these not included in the models? Could the authors perhaps clarify in the methods section which potential confounders where included in the models (and why e.g. BMI and sleep problems were not included, if this is the case).
*Response: Thank you for pointing out this omission. We have edited the text on page 7 to now clearly explain: “Regression models were used to explore the relationship between i) PA at 42y and cognitive function at 50y; ii) PA at 42y and NETCRF at 45y; and iii) NETCRF at 45y and cognitive function at 50y. Models were first unadjusted and then adjusted for baseline confounders listed above (the NETCRF―cognition association was additionally adjusted for PA at 42y, and intermediate confounders measured at 45y: BMI, PA level, sleep problems and self-rated health over the previous 12 months). Importantly, for the PA at 42y and cognitive function at 50y association, we did not adjust for NETCRF or the intermediate confounders. This is because adjusting for these intermediate variables would attenuate the overall effect, which is the estimand of interest here.” We have also updated the analysis in Table 2 (Model C in particular) to reflect the fact that we now appropriately adjust the NETCRF―cognition association for the intermediate confounders mentioned above, and we have also updated the corresponding footnote.*

Supplementary
- DAG: I am not entirely convinced when it comes to the direction of the association between sleep problems at 45 years of age and CRF at the same age. It has been suggested that better CRF may improve sleep (see, for instance: Ernstsen et al., 2023 DOI: 10.1016/j.mayocp.2022.08.013; Dishman et al., 2015 DOI: 10.1249/MSS.0000000000000506), making sleep quality a potential mediator in the CRF-cognitive function association, rather than a confounder. Although it is unlikely that adjustment for sleep problems was the reason for the lack of association between NETCRF and cognitive function in this study, I think the authors should reconsider the inclusion of sleep problems as a confounder.

*Response: We agree with the reviewer that it is unlikely that adjustment for sleep problems was the reason for the lack of association between NETCRF and cognitive function in our study. This is supported by data presented in Table 2 showing that the NETCRF and cognitive function association attenuates to the null when adjusting for baseline confounders (Model B). However, the reviewer is correct that the sleep-CRF association is difficult to disentangle when measured at the same age. We have therefore added to the discussion the following text with appropriate references (page 19): “Moreover, our intermediate confounders (e.g., sleep problems) were measured at the same time as our mediator/moderator (NETCRF) and we acknowledge that the direction of association between factors measured contemporaneously are not always straight-forward to disentangle (in particular for sleep problems)[55-57].”*

**Reviewer #2**

I enjoyed reading this submission, and really looked forward to the mediation/moderation analysis. On accepting the review, I very much was looking forward to how fitness was going to be justified in the background, as I admit, I don’t see why a relationship should exist. From this perspective, I was disappointed to find that little justification for looking at this relationship was offered. Given the findings were consistent with my own biases, that is no mediation/moderation effect, I can see how this could find a place in the literature supporting many others' views. But I do not feel easy with how this paper could be accepted in the literature, and I wonder if it is the way the data and analysis were used, I don’t believe there was a chance for any existing effect to be found. And I do not believe there is much recognition for these limitations. I am not sure what data you have available within this data set, but my concerns are around the self-report nature of PA, and the comparison of the existence of PA within the self-report data (at least once per week), or not (no record per week). Surely if fitness was to have a mediating or moderating effect, there would need to be a consideration of a dose-response, rather than the dichotomised approach of PA being present or not. These general mechanistic considerations seem much more important than a particular analytical approach, and so my major concerns with this manuscript centre there. I do however think that showing the existence of PA impacting later-life cognition is more important than recognised in the manuscript. I have made further specific comments as I read through the manuscript. Whilst the broad statistical approach makes sense to me, I cannot offer commentary on the specifics.

*Response: Thank you for your feedback. In response to the individual comments below, we explain how we address all raised concerns including a justification as to why fitness was considered a mediator/moderator (with appropriate references to potential mechanisms). We have also expanded the limitations section and provide more data on potential dose-response associations to justify the activity dichotomy we have used. We believe these additions have strengthened our manuscript.*

Background: Can there be a clearer justification of why CRF may have a mediating/moderating effect? I don’t really see why this should exist. There is nothing I can think of that could suggest this, apart from maybe a link to vascular health.. but I feel that is a stretch. A stronger argument may be saying that others have suggested it (if they have?) and so we sort to test it. Mechanistically though (biologically, not statistically), I struggle to see how the relationship should exist in that way, whereas the reverse argument is much clearer and obvious. The moderating argument also suggests that CRF may enable greater PA… but if this was the case, then analysing data that extends beyond a dichotomy of at least once per week, or less than once per week is surely warranted. So given the justification, it does not feel like the methods follow through with the current methodology.
*Response: We have edited both the introduction and discussion to address this point with appropriate references where others have suggested mediation and/or moderation, as well as to underlying biological mechanisms as appropriate. Specifically:*

- *to highlight putative relationships between exposure (physical activity), mediator (CRF) and outcome (cognition), we now state in the 2^nd^ paragraph of the introduction: “Higher levels of PA are associated with lower risk of all-cause dementia(4, 5), cognitive decline(6) and better later-life cognition(7). However, pathways underpinning the PA―cognition association are unclear. While cardiovascular(8) and mental(5) health may be involved, an additional pathway may include cardiorespiratory fitness (CRF). CRF refers to the capacity of the circulatory and respiratory systems to supply oxygen, and the ability of muscle tissue, to utilise it(9). PA, particularly aerobic activity, has established beneficial effects on CRF(10) and evidence suggests that higher CRF is associated with greater subsequent cognitive function(11). Thus, CRF may have a mediating role in the PA―cognition association.”*
- *In terms of lack of evidence regarding CRF being a mediator of the physical activity―cognition association, we speculate in the discussion on other potential mechanistic pathways (page 18): “If positive effects of mid-life PA on cognitive function are not due to increases in fitness, then what may be the mode of action? Several mechanisms have been proposed and include a reduced likelihood of vascular diseases and improvements in cerebral perfusion(44-46), stimulation of growth factors (e.g., brain-derived neurotrophic factor(47)), and/or the downregulation of oxidative stress and inflammatory responses(48). Alternatively, PA may positively affect cognitive function, independent of the movement component of exercise, due to the cognitively stimulating aspects of PA, such as eye-hand coordination, visuospatial memory, self-motivation(49), planning and social interaction(50, 51).”*
- *With respect to the putative effect modification of the physical activity―cognition association by CRF, we now state in the 2^nd^ paragraph of the introduction: “However, the link between PA and CRF is likely to be dynamic, because, for example, those with higher levels of fitness might participate in PA more often and/or at greater volumes/intensity. Thus, in addition to CRF potentially mediating the PA―cognition association, CRF may also moderate this association, with the association differing depending on fitness levels. However, evidence is mixed and may be specific to certain cognitive domains. For example, some studies suggest that the effect of PA on memory is stronger in fitter individuals(12), whilst others find the positive effect of PA on processing speed is stronger in less-fit individuals(13).”*
- *In terms of the mixed evidence for CRF being a moderator of the physical activity―cognition association, we state in the discussion (page 18): “In trying to explain the apparent lack of a PA-CRF-cognitive outcome mechanism of action, Young et al (2015) suggested that PA effects may operate in certain subgroups of the population only, such as those starting from a lower baseline of CRF, i.e., a moderating effect may exist between PA and CRF(39)…. Previous studies have reported mixed findings regarding the interactive effect of fitness on the relationship between PA and cognitive outcomes, with some observing evidence of a greater benefit of PA in those with higher fitness(12), lower fitness(13), or no effect(52).”*
- *Finaly, with regard to analysing data that extends beyond a dichotomy of at least once per week, or less than once per week, please see response to “Why was the exposure (PA frequency) dichotomised?” below.*

Methods: I am not sure I agree that there is strong validity of the prediction equations from the Jurca et al 2005 paper. It would be better to report the results they actually got. Could you also highlight differences in how the variables used in the prediction equations between those used in the Jucra paper, and in this manuscript? From what I can see, it does not sound like the self-report measures were similar, nor if measures such as resting heart rate were similarly assessed.

*Response: We have now edited the text on page 5/6, to state the validity of the prediction equations reported in the Jurca et al 2005 paper. It now states: Of the three NETCRF algorithms originally developed(21), we selected the algorithm developed[22], and previously used[23], in UK populations. This algorithm has shown a multiple correlation of 0.76 against exercise testing estimated CRF. It also has a high level of cross-validity (0.71–0.80) when applied in samples other than the one it was developed in[21].*

*We now also explain in the text, how physical activity at 45y was measured (page 5): “A modified version of the European Prospective Investigation into Cancer and Nutrition Physical Activity Questionnaire (EPIC-PAQ) was used to assess leisure-time PA(20).” And how, similar to previous studies (see Stamatakis et al. European Heart Journal 2013;34(10):750-8), the physical activity coefficients were mapped onto the prediction equation (page 6): “The PA coefficients were based on adherence to contemporaneous guidelines assessed using EPIC-PAQ, and mapped onto the prediction equation, similarly to previous studies(23): 0.0 for inactive during leisure-time; 0.29 for active, but not meeting guidelines; and, 1.21 for meeting guidelines (i.e., at least 150minutes/week moderate-intensity or 75minutes/week vigorous-intensity PA)”.*

*We also provide references to the standard protocols used to measure weight and height and provide details of the device used to measure resting heart rate (page 5): “In 2003 when participants were 45y (range 44y-46y), weight and height were measured using standard protocols(19); body mass index (BMI; kg/m^2^) was calculated. After a few minutes rest, three measurements of resting heart rate (RHR, beats/minute) were obtained (taken one minute apart) using an automated device (Omron 907 blood pressure monitor, Omron Healthcare, UK); mean RHR was calculated.”*

Why was the exposure (PA frequency) dichotomised? What is the rationale for this? It seems more detail is available. What issues are there in the analysis if the same measures (self-report PA) are used in both the PA and NETCRF? Can they truly be used to evaluate if one mediates/moderates another in the prediction of a third (independent) measure?

Response: T*he rationale for dichotomising the exposure into activity frequency of at least once per week was a pragmatic one. We did not have information at 42y on activity duration and intensity. Therefore, a more nuanced measure of activity at 42y was not possible. We acknowledge this in the discussion by now stating (page 19): “Importantly, our binary exposure, PA at 42y, was based on self-report and limited to leisure-time activity, rather than total activity, which would include other domains (e.g., occupation). It may be subject to measurement error and bias and, due to data limitations, we were unable to consider duration and intensity of PA at 42y. Nonetheless our 42y PA measure and analysis focusing on a counterfactual reality, whereby the least active group in the population (active less than once per week) become active (at least once per week; regardless of whether or not they meet recommended guidelines) has value,* *as supported by dose-response associations with mortality showing greatest benefit at the lower end of the physical activity spectrum (i.e., going from ‘nothing to ‘anything’)[58]. Additionally, previous findings of our activity measure (e.g. with blood pressure[59]) provides construct validity and elsewhere has been associated with important health outcomes including mortality[60, 61].”*

*Nonetheless, we acknowledge the reviewers concern and provide in the table below associations for the mean difference in NETCRF (45y) and overall cognition function (50y) by categories of physical activity frequency at 42y. While there is evidence of a potential dose response relationship between activity frequency and NETCRF, no such evidence is found for activity frequency and cognitive function. Regarding the latter, and due to the lack of granularity in the activity data available (i.e., no data on 42y activity time and intensity), we are unable to state with confidence whether the lack of a dose-response association should be presumed accurate or is inaccurate due to the ill-defined nature of the multiple physical activity frequency categories. Thus, we believe that presenting a dose-response effect of activity relies heavily on unverifiable assumptions. Therefore, as now explained with the above edits to the discussion, we opted to focus on the extreme of inactivity, i.e., changing from doing nothing to something, as this is both informative and reliable.*

*Mean difference in NETCRF (45y) and overall cognition function (50y) by physical activity frequency at 42y*

|  | Mean difference in NETCRF (METS)(45y)  (95% CI) | | Mean difference in overall cognition function z-score (50y)  (95% CI) | |
| --- | --- | --- | --- | --- |
|  | *Males* | | | |
| *Physical activity frequency at 42y* | *Model A* | *Model B* | *Model A* | *Model B* |
| *<Once per week* | Reference | Reference | Reference | Reference |
| *Once per week* | 0.35 (0.24, 0.47) | 0.34 (0.19, 0.49) | 0.16 (0.10, 0.21) | 0.05 (-0.02, 0.12) |
| *2-3 times per week* | 0.59 (0.48, 0.71) | 0.47 (0.33, 0.62) | 0.17 (0.12, 0.23) | 0.05 (-0.02, 0.11) |
| *4-7 times per week* | 0.64 (0.52, 0.75) | 0.53 (0.38, 0.68) | 0.13 (0.08, 0.19) | 0.06 (-0.01, 0.12) |
|  | *Females* | | | |
| *<Once per week* | Reference | Reference | Reference | Reference |
| *Once per week* | 0.42 (0.29, 0.56) | 0.32 (0.15, 0.49) | 0.11 (0.06, 0.17) | 0.02 (-0.05, 0.08) |
| *2-3 times per week* | 0.64 (0.51, 0.77) | 0.52 (0.36, 0.68) | 0.15 (0.09, 0.20) | 0.02 (-0.05, 0.08) |
| *4-7 times per week* | 0.70 (0.58, 0.82) | 0.66 (0.51, 0.81) | 0.07 (0.02, 0.20) | 0.02 (-0.03, 0.09) |

Model A: unadjusted; Model B: adjusted for baseline confounders (socioeconomic position at birth, sports participation at 16y, cognitive function at 11y, academic attainment at 33y, smoking status at 42y, alcohol consumption at 42y)

*Regarding the use of physical activity at 42y as an exposure (that causes CRF) and physical activity at 45y as a predictor of NETCRF, conceptually and statistically this is acceptable. We now explicitly state this on page 7: “Of note, the causal diagram in S2 Fig, aims to illustrate underlying causal networks underpinning the relationship between 42y PA and 50y cognitive function. This is distinct from the prediction equation for NETCRF at 45y described above. As such, variables that perform well in prediction models may not be causal[27]. Similarly, some variables e.g. BMI can perform well in predicting, and also, cause CRF.” Also noteworthy is that in the calculation of NETCRF, 45y activity is not the strongest loading factor (ranging from 0 to 1.21) when compared to, for example, sex (ranging from 0 to 2.78).*

*On rereading the manuscript, there may have been some ambiguity regarding the measurement and timing of the physical activity measures used as exposure (at 42y) and as predictor of NETCRF (at 45y). It is important to register that activity was measured differently at 42y (as a frequency) and at 45y (using a modified version of the European Prospective Investigation into Cancer and Nutrition Physical Activity Questionnaire). Our edits mentioned in response to previous queries, as well as the subscripts now used in the NETCRF equation, should make this distinction clearer.*

How sensitive do we think these cognitive measures are? Could that be the reason why a relationship is not observed? Equally, how sensitive do we think the self-report PA is? These do not appear to garner much attention in the discussion.
*Response: We have added the following text to the limitations section of the discussion to acknowledge this point in relation to cognition (page 19/20): “While the cognitive function measures used have been adopted in other large-scale epidemiological studies(25, 63) and have been shown to be predictive of incident dementia(26), they only reflect fluid cognition. As such, using a measure of crystallised cognition may have resulted in different associations being observed.”*

*With regard to the utility of self-reported (and dichotomised) activity, please see the edits mentioned in the above response that acknowledge these limitations.*

BMI (yrs) also appears to be used both within the creation of the mediator variable, and then appears as a potential confounder as well. Same with PA level (45 yrs), are these variables included multiple times in the analysis?

*Response: We now make explicit distinction between causal links and prediction (with appropriate references) by stating in the methods (page 7): “Of note, the causal diagram in S2 Fig, aims to illustrate underlying causal networks underpinning the relationship between 42y PA and 50y cognitive function. This is distinct from the prediction equation for NETCRF at 45y described above. As such, variables that perform well in prediction models may not be causal[27]. Similarly, some variables e.g. BMI can perform well in predicting, and also, cause CRF.”*

Can you clarify what missing data was imputed? And how this was done? It is not clear to me how some of it was calculated (e.g., BMI, PA) given some of this data is already in the imputation model.

*Response: We have edited the text on page 7 to explain that we follow current guidelines to impute data by chained equations (and this is how/why variables which have missing variables in them are themselves included in the imputation model). To ensure we write succinctly, we now provide a reference to best practise, which we followed in our imputation. The text also explains that we impute all missing data presented in Table 1, we now state: “To address missingness in data presented in Table 1, we used multiple imputation by chained equations following recommended guidelines[30]. We assume the data are missing-at-random and strengthen this assumption by including in the imputation models all variables described above (i.e., 42y PA, 45y NETCRF, 50y cognitive function and potential confounders), as well as childhood internalizing and externalizing behaviours and cognitive ability which have been shown to predict missingness in follow-up(17). The number of imputations (n=30) required to achieve convergence of parameter estimates was determined as 100×fraction missing information(31).”*

Table 1: sports participation – I don’t think that is mentioned earlier in the document. Is this used as a potential confounder?

*Response: Thank you for this comment. The omission of this variable in the description of baseline confounders was a mistake which we have now rectified.*

Discussion
In relation to the second paragraph, could it be that PA becomes more important as we get older, rather than accumulation of PA per se?
*Response: Yes, good point. We now state at the end of this paragraph: “Alternatively, similar to associations between physical activity and depressive symptoms[36], the magnitude of the physical activityꟷcognition association may vary by life-stage.”*

Third paragraph – given the dichotomy of the analysis – is this really a fair conclusion? That the impact is sex specific? Could it just be that the dose comparison between the two groups is different? But this is ignored in the current analysis?

*Response: Please see previous responses where we explain edits to the text regarding the dichotomy of the activity variable. In addition to those edits, we also add to this paragraph (page 17/18): “Discrepancies between findings could be due to differences between the life-stage examined or the measure of physical activity used (for example, the dichotomy of activity examined here, could have different implications by sex).”*

Strengths – “to more accurately reflect likely processes in the real world”… given my comments earlier, particularly in relation to the likely dose effects of PA, I don’t see how this claim can be made. I don’t disagree it may be a better way of statistically understanding relationships, but without it being based on a strong rationale, this statement seems grossly inappropriate.
*Response: We have edited to emphasise the statistical modelling advantages of our approach, the sentence now reads: “For example, our use of a four-way decomposition analysis, has allowed us, for the first time, to more accurately model real world processes, thus avoiding potential biases inherent in simpler methods.”*

References: A few inconsistencies in the referencing… e.g., use of capitals etc.

*Response: We have corrected these inconsistencies.*
